# Wound closure after total knee replacement: Comparison between staples and sutures

**DOI:** 10.12669/pjms.38.ICON-2022.5782

**Published:** 2022-01

**Authors:** Mansoor Ali Khan, M. Waseem Memon, Amin Chinoy, Salman Javed, Rahil Barkat, Ahsun Jiwani

**Affiliations:** 1 Dr. Mansoor Ali Khan FCPS. Professor, Department of Orthopedics, The Indus Hospital, Karachi, Pakistan; 2 M. Waseem Memon, FCPS. Junior Consultant, Department of Orthopedics, The Indus Hospital, Badin, Pakistan; 3 Amin Chinoy, FRCS. Professor, Department of Orthopedics, The Indus Hospital, Karachi, Pakistan; 4 Dr. Salman Javed, FCPS. Junior Consultant, Department of Orthopedics, The Indus Hospital, Karachi, Pakistan; 5 Mr. Rahil Barkat, MSc. Junior Epidemiologist, The Indus Hospital, Karachi, Pakistan; 6 Mr. Ahsun Jiwani, MSc. Junior Epidemiologist, The Indus Hospital, Karachi, Pakistan

**Keywords:** Staples, Sutures, Total Knee Replacement

## Abstract

**Background and Objective:**

Total Knee Arthroplasty is a commonly performed procedure for arthritic knees. Preventing complications is of utmost importance for good functional outcomes and preventing morbidity. Wound closure after the procedure is as important as the replacement aspect of surgery.The objective of this study was to provide subjective and objective evidence of better closure technique and material; we conducted the study so that the outcome of TKA can be further improved

**Methods:**

We conducted a randomized trial at The Indus Hospital, Karachi, from December 2018 to June 2020. All patients from age 40 to 70 years who underwent total knee arthroplasty were included in the study. The wound of one knee was closed with Polypropylene (Prolene) sutures, and the other with staples. The wound was assessed independently by two assessors using Hollander’s score; lower score means a worse outcome. All data was entered and analyzed using STATA version 16.

**Results:**

Thirty patients who underwent bilateral total knee replacement were included in the analysis, among which 71.8% were female. The average age of participants was 57.3 (± 7.5) years. The mean incision length on the right knee was 17.6 ± 1.1 cm, while on the left the incision length was 18.3 ± 1.2 cm. Overall, the mean Hollander score was significantly different among participants in the sutures and staples group in both the right (p-value=0.001) and left knees (p-value=0.001). The score was significantly higher in knees closed with sutures as compared to staples. Also, the mean Hollander score is significantly higher in females than males in both the right knee (B=0.56, p-value=0.049) and the left knee (B=0.38, p-value=0.044).

**Conclusion:**

The study has shown that Hollander’s score was significantly higher in knees closed with sutures as compared to the patients in whom staples were used for wound closure.

## INTRODUCTION

Total knee arthroplasty (TKA) is one of the most commonly performed procedures as a definitive treatment for arthritis.^1^ Even though outcomes of TKA have improved considerably in recent years, few complications are still common. There are several complications of TKA which include bleeding, thromboembolism, neural deficit, vascular injury, deep joint infection, implant loosening, patellofemoral dislocation, tibiofemoral dislocation, and reoperation.^2^,^3^

The post-operative outcomes are regulated by the following factors: preoperative education, an understanding of rehabilitation procedures, maintenaning operative protocols, and postoperative wound care. Wound closure and its management is one of the most important aspect of surgical procedures. Poor technique or inappropriate wound closure can lead to surgical site infections, which can be devastating following TKA. It sabotages patients’ recovery and significantly elevates the morbidity rate which may ultimately lead to treatment failure.^4^-^6^ Various wound closure techniques and materials are used for skin closure, such as sutures, staples, adhesive compounds, etc.^7^

The use of sutures for wound closure is an important part of a surgical procedure. Sutures helps to control bleeding, provides better approximation, and are a time-effective.^8^ However, for closing deeper layer, braided sutures are considered to have fewer postoperative complications.^9^ Recently, skin closure using staples has been considered a preferred choice. This is because they provide the fastest closure with a lower incidence of infection.

Although few studies have been published showing the superiority of one method/material over the other, it is mostly the surgeon’s preference which closure method and material are used. An important aspect of wound closure is that the wound should be closed tension-free with watertight everted skin-edges, which ultimately is very important for wound healing.^10^ We, therefore, aimed to provide subjective and objective evidence of better closure technique and material; we conducted the study so that the outcome of TKA be further improved.

## METHODS

A randomized controlled trial was conducted at the Orthopedics Department of The Indus Hospital, Karachi, from December 2018 to June 2020. We recruited 30 patients undergoing bilateral TKA. Patients aged between 40-70 years undergoing primary TKA for osteoarthritis, rheumatoid arthritis, or post-traumatic arthritis were included. Patients having previous skin, neuromuscular, or connective tissue disorder, and those taking steroids or with a BMI >30 were excluded. All the patients were recruited at the Orthopedics outpatient clinic after assessing their eligibility criteria. Informed consent was obtained following the guidelines of the Institutional Review Board after obtaining an IRB approval for the study (Ref. No. IRD_IRB_2018_06_006; dated Dec. 12, 2019).

Baseline data regarding the patient’s age, gender, height, weight, ASA grade, comorbidities, primary diagnosis, incision length (measured by standardized sterile measuring scale), and duration of surgery were recorded. If a wound was found to be infected or had discharge, a wound culture and sensitivity test were done. All patients participating in the trial followed a standardized care pathway for surgical wounds. The surgeries were performed by one of the senior surgeons of the orthopedics department. The patients were daily followed until discharge. A detailed wound assessment of all the participants was performed on 3^rd^, 7^th^, 15^th^, 30^th^, and one year, postoperatively by an independent senior surgeon from the orthopedics department.

### Blinding and Randomization:

This trial was an open-label trial. The patients, surgeon, and the study team couldn’t be blinded to the study intervention due to the different nature of visible sutures used on the skin. Randomization was done, and envelopes were prepared using SNOSE protocol, i.e., they were sequentially numbered, opaque sealed envelopes.^11^ Before opening the envelope, the primary investigator wrote the patient’s medical record number, date and signed the envelope. The envelope contained carbon paper which transferred the handwritten data on the allocation paper inside. The patients were randomized into two intervention groups. In the interventional ARM-1, the wound closure of the right knee was done using staples, and the wound closure of the left knee was done with polypropylene (prolene) sutures. In the interventional ARM-2, the wound closure of the right knee with prolene sutures and wound closure of the left knee was done with staples.

Study products were prolene sutures and staples. Prolene sutures are non-absorbable, sterile surgical sutures composed of an isotactic crystalline stereoisomer of polypropylene. After completion of the procedure, deep tissues were closed with a subcuticular prolene suture. Specialized staples are used in surgery in place of sutures to close skin wounds. After completion of the procedure, deep tissues were closed with absorbable braided sutures, and then the skin was closed using staples.

Data were analyzed using STATA Version 16.0. For continuous variables, mean and standard deviation were calculated, and for categorical variables, frequencies and percentages were determined. Mean Hollander score was computed at 3^rd^, 7^th^, 15^th^, 30^th^, and 1-year follow-up along with its standard deviation. Mixed model linear regression was used to determine the impact of sutures and staples on wound healing after total knee replacement. A separate analysis was done for the right knee and left knee to understand whether the impact of two groups on wound healing is constant on each knee or not. To assess the significance, the cut-off of the p-value was kept at 0.05.

## RESULTS

Thirty patients who underwent bilateral total knee replacement were included in the analysis. The characteristics of study participants is shown in Table-I. The majority of participants were females, i.e., 71.8%. The average age of participants is 57.3 (± 7.5) years. The numbers of knees closed by staples were the same as the numbers of knees closed by using sutures, i.e., 30 in each group. Twenty-seven patients were obese (90%). Eleven patients (52.4%) had diabetes, while Twenty-three patients (85.2%) had hypertension. The mean incision length on the right knee is 17.6 ± 1.1 cm, while the mean incision on the left length is 18.3 ± 1.2 cm.

**Table I:** Demographical information of Patients n=30.

**Age**	
Mean ± SD (Years)	57.3 ± 7.5
**Gender**	
Male	10 (33.3%)
Female	20 (66.7%)
**ASA Level**	
I	06 (20%)
II	21 (70%)
III	03 (10%)
**Hypertension (n=27)**	
Yes	23 (85.2%)
No	04 (14.8%)
**Diabetes (n=21)**	
Yes	11 (52.4%)
No	10 (47.6%)
**Albumin Level**
Mean ± SD	3.9 ± 0.33
**Body Mass Index (BMI)**	
Normal	01 (3.3%)
Overweight	02 (6.6%)
Obese	27 (90%)
**Open Surgical Procedure on the Same Site**	
Yes	02 (6.7%)
No	28 (93.3%)
**Infection in any other body part**	
No	30 (100%)
**Incision Length on Right Knee**	
Mean ± SD	17.6 ± 1.1
Min-Max	16-20
**Incision Length on Left Knee**	
Mean ± SD	18.3 ± 1.2
**Hollander Scores**	
**Right Knee**	
3^rd^ post-op day	4.5 ±1.3
7th post-op day	4.5 ±1.3
15^th^ post-op day	4.6 ±1.2
30^th^ post-op day	4.8 ±1.1
1 year follow-up	4.9 ±0.6
**Left Knee**	
3^rd^ post-op day	3.2 ±1.3
7th post-op day	3.6 ±1.3
15^th^ post-op day	3.8 ±1.2
30^th^ post-op day	3.9 ±1.1
1 year follow-up	3.9 ±0.6

The mean Hollander scores of patients at different time intervals and effect of suture and staples on both right and left knee are presented in Table-II. On the right knee, the mean Hollander score was highest after one year of surgery, i.e., 4.06 followed by the 30th postoperative day (4.8±1.1). On the left knee, the mean Hollander score on the 3rd postoperative day was 3.2 (±1.3). On the 7th postoperative day, the mean score was 3.6 (±1.3). The mean Hollander score was highest after one year of surgery, i.e., 3.9 ±0.6.Overall, the mean Hollander score was significantly different among participants in the sutures and staple group in both right knees (p-value=0.001) and left knee (p-value=0.001). . In the right and left knee, the overall difference of mean Hollander score between the two groups is 2.6 and 1.96, respectively. Gender was significantly associated with the mean Hollander score. The mean Hollander score was significantly higher in females than males in both right knee (B=0.56, p-value=0.049) and left knee (B=0.38, p-value=0.044).

**Table II:** Effect of Study groups on Hollander Score (Right Knee and Left Knee).

	Right Knee		Left Knee	

Variable	B (95% CI)	p-value	B (95% CI)	p-value
**Treatment**				
Staples	Reference			
Sutures	2.26 (1.31-2.90)	0.001*	1.96 (1.43-2.50)	0.001*
**Time**				
Day 3	Reference			
Day 7	0.30 (-0.07-0.69)	0.119	0.28 (-0.05-0.61)	0.1
Day 15	0.52 (0.13-0.90)	0.001*	0.46 (0.13-0.80)	0.006*
Day 30	0.81 (0.42-1.19)	0.001*	0.65 (0.32-0.98)	0.001*
Day 365	1.81 (1.38-2.24)	0.001*	1.5 (1.15-1.84)	0.001*
**Gender**				
Male	Reference			
Female	0.57 (0.002-1.13)	0.049*	0.61 (0.008-1.19)	0.045*

* Significant at p-value<0.05.

## DISCUSSION

Total knee replacement has evolved over time with respect to surgical techniques^12^ Soft tissue handling is of equal importance in its success as is the proper implant placement. Early rehabilitation post-surgery is dependent on optimal wound healing. Hence wound closure material and technique carry great importance.

Studies have shown that wound closure at flexed position can allow more flexion and better rehabilitation.^13^ Although the closure position is surgeon-dependent, but is of paramount importance. Similarly, the material used for closure also dictates the outcomes; the functional outcome aong with the cosmesis.

In our study, we aimed to determine the best outcome of the material for skin closure after total knee replacement. Since, significant knee flexion and extension rehabilitation therapies starts after surgery, would healing has significant value. For this purpose, the same patient underwent different closures in different knees, which helped us to exclude the patient-related factors. We used the Hollander Wound evalution scale, which is a validated scale and has been previously used in the assessment of wounds in other surgeries. Wound-related complications in our study were considerably less than previously reported.^14^-^16^

Yuenyongviwat et al. had reported no overall difference between the two groups (suture versus staples).^6^ However, Yuenyongviwat et al. used sutures and staples in the same knee (upper wound closed with staples and lower have by sutures), hence had a higher rate of infection then our study. In our study, sutures were found to have better results over staples as the Hollander score for females and those closed with sutures was reported to be higher.^6^ Khan et al. used skin staples, subcuticular sutures, and 2-octyl cyanoacrylate for closure of wounds in hip and knee replacements and found no difference in outcome.^17^ Our study reported different outcome than both Yuenyongviwat et al and Khan et al. however, they had followed the patient for six weeks while we have reported our final outcomes after one year follow up.

Hlubek et al. had different ourcomes from our study and preferred staples over sutures however, they reported higher and severe infection rate with staples.^14^ Kerbs et al. reported no difference in the two methodes of skin closure.^9^ The majority of previous authors had compared suturing time and pain in removal with more interest than the overall functional and cosmetic aspects.^17^-^19^ We did not take into account the suturing time as this could have affected outcomes. Newman et al. reported a 9% complication rate with sutures, which included superficial and deep infections. This proved to be different from our findings as no significant infection was reported in both groups. However, their findings in terms of cosmesis were similar with staples and sutures.^20^

Hollander scoring also pointed towards the overall satisfaction with respect to cosmesis. In the randomized trial by Clayer et al. on hip surgeries, the better cosmesis was noted with sutures which were similar to our study findings, but in knee joint compared to hip the incisions vary in size and direction and the amount of stress on the wound during moving is different.^21^ Similarly, Singh et al. found similar results to us, indicating significantly less wound discharge and erythema with the use of sutures.^15^

The wound was also assessed independently by the assessors at the same time, which added more reliability to our results. Our follow-up was of one year, which was the strength of our study. The reason of better outcomes with continuous running sutures is also explained by a study by Wyles et al.^21^ which reported running sutures maintained better perfusion to the skin, hence leads to better healing and cosmesis.

### Limitation of the study:

Our study had a small sample size, and although nor the patient or the assessor was blinded, using different techniques of closure on the same patient helped us remove all patient-related factors. We had the closures done in a similar way by the same surgeon, which removed any bias. We did not take into account the position of wound closure, which according to the literature might also affect the outcomes. We also did not consider the removal of suture kind staples technique as that can also cause pain and morbidity to the patient. We recommend a trial with a larger sample size to determine the best method of closure so the patient outcomes could further be improved.

## CONCLUSION

The study has shown that Hollander’s score was significantly higher in knees in which sutures were used as compared to the patients in which staples were used for wound closure. Skin staples had an advantage over prolene sutures in terms of operative time, but on the other hand, they are more difficult to remove than prolene stitches.

### Authors` Contrubtion:

**MAK:** Conceived and designed the study.

**MWM:** Data collection and manuscript writing and responsible for the integrity the manuscript.

**MAC:** Editing and critical review of the manuscript.

**SJ:** Prepared the manuscript and is responsible for the integrity of the study.

**RB:** Performed the analysis and editing of the manuscript.

**AJ:** Performed the analysis.
